# Characterization of the genetic switch from phage ɸ13 important for *Staphylococcus aureus* colonization in humans

**DOI:** 10.1002/mbo3.1245

**Published:** 2021-10-14

**Authors:** Camilla S. Kristensen, Anders K. Varming, Helena A. K. Leinweber, Karin Hammer, Leila Lo Leggio, Hanne Ingmer, Mogens Kilstrup

**Affiliations:** ^1^ Department of Biotechnology and Biomedicine Technical University of Denmark Lyngby Denmark; ^2^ Department of Chemistry University of Copenhagen Kobenhavn Denmark; ^3^ Department of Veterinary and Animal Sciences University of Copenhagen Kobenhavn Denmark

**Keywords:** bacteriophage, genetic switch, lysogeny, ɸ13, phi13, prophage, repressor, *Staphylococcus aureus*

## Abstract

Temperate phages are bacterial viruses that after infection either reside integrated into a bacterial genome as prophages forming lysogens or multiply in a lytic lifecycle. The decision between lifestyles is determined by a switch involving a phage‐encoded repressor, CI, and a promoter region from which lytic and lysogenic genes are divergently transcribed. Here, we investigate the switch of phage ɸ13 from the human pathogen *Staphylococcus aureus*. ɸ13 encodes several virulence factors and is prevalent in *S. aureus* strains colonizing humans. We show that the ɸ13 switch harbors a *cI* gene, a predicted *mor* (modulator of repression) gene, and three high‐affinity operator sites binding CI. To quantify the decision between lytic and lysogenic lifestyle, we introduced reporter plasmids that carry the 1.3 kb switch region from ɸ13 with the lytic promoter fused to *lacZ* into *S. aureus* and *Bacillus subtilis*. Analysis of β‐galactosidase expression indicated that decision frequency is independent of host factors. The white “lysogenic” phenotype, which relies on the expression of *cI*, could be switched to a stable blue “lytic” phenotype by DNA damaging agents. We have characterized lifestyle decisions of phage ɸ13, and our approach may be applied to other temperate phages encoding virulence factors in *S. aureus*.

## Graphical abstract

Bacteriophage ɸ13 belongs to a group of phages that carries virulence factors and colonizes human strains of *Staphylococcus aureus*. Knowledge about the mechanism behind the genetic switch that decides between lysogeny (prophage integration) or lysis (bacterial killing) is important for combating *S. aureus* infection. Using the switch region from ɸ13, we have created reporter plasmids that mimic the decision phase and can detect prophage‐inducing conditions.

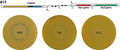

## 1 INTRODUCTION

*Staphylococcus aureus* is a Gram‐positive, opportunistic human pathogen causing millions of infections worldwide each year, ranging from skin and soft tissue infections, food poisoning, endocarditis, and respiratory tract infections to bacteremia, amongst many others (Liu, 2009; Lowy, 1998; Tong et al., 2015). The severity of the infections varies from mild to life‐threatening, largely determined by the various virulence factors contained in the infecting strain.

A number of important virulence factors are encoded by *S. aureus* prophages that stably reside in the bacterial genome (Xia & Wolz, 2014). Most clinical strains of *S. aureus* are lysogens and carry between one and four prophages that encode important toxins, such as Panton‐Valentine leucocidin (PVL) and enterotoxins (Ingmer et al., 2019). Some of the most common prophages in human isolates of *S. aureus* belong to the Sa3int group. These phages integrate into the *hlb* gene of *S. aureus* and express an immune evasion cluster encoding several virulence factors promoting colonization (Coleman et al., 1991; Xia & Wolz, 2014). As the beta‐hemolysin encoded by *hlb* is also a virulence factor (Huseby et al., 2007; Katayama et al., 2013), this negative conversion appears counterintuitive, but Sa3int phages have been shown to excise and remain as pseudo‐lysogens, allowing expression of both the beta‐hemolysin and the virulence factors encoded by the phage (Goerke et al., 2006; Katayama et al., 2013; Salgado‐Pabón et al., 2014).

Temperate phages have a dual lifecycle involving either lytic replication and production of active phages or being inserted in and replicated with the bacterial chromosome. The decision between these lifestyles is made by a phage‐encoded (epi)genetic switch. Phage λ, targeting *Escherichia coli*, offers the classical example of such a switch, where a complex decision phase determines from which of the two involved promoters, P_R_ or P_RM_, transcription occurs. Expression from the P_RM_ promoter produces the phage repressor CI, whereas expression from P_R_ allows the production of the Cro repressor, as well as other gene products needed for producing active phage particles (Casjens & Hendrix, 2015; Oppenheim et al., 2005). Stable expression from the P_RM_ promoter is needed for the maintenance of the λ chromosome into the *E. coli* chromosome. It also produces a high concentration of the CI repressor, which is required for activation of the P_RM_ promoter and tight repression of the P_R_ promoter in the integrated prophage and any incoming λ phages, rendering the lysogenic bacterium immune to secondary attacks. The decision and integration of phage λ are not solely dependent upon the phage‐encoded factors, as *E. coli* host factors also affect this process (Grodzicker et al., 1972; Herman et al., 1993; Kihara et al., 2001; Roucourt & Lavigne, 2009). The escape of phage λ from the lysogenic state can be initiated by autocleavage of the CI repressor. In a process analogous to autocleavage of the LexA repressor, CI is bound to activated RecA filaments polymerized on single‐stranded DNA during the SOS DNA damage response, leading to activation of CI auto‐peptidase activity (Atsumi & Little, 2006; Little, 1984; Oppenheim et al., 2005). A quite different switch mechanism is used by the TP901‐1 phage infecting the Gram‐positive bacterium *Lactococcus lactis* (Madsen et al., 1999) which shares extensive homology to the ɸ13 switch (Pedersen et al., 2020). Here, a CI repressor, expressed from the lysogenic promoter P_R_, represses the lytic P_L_ promoter. A small repressor (MOR, for modulator of repression) expressed from the P_L_ promoter represses the P_R_ promoter; however, it does require CI as a co‐repressor. In addition MOR functions as an antirepressor against CI (Pedersen & Hammer, 2008).

As a member of the staphylococcal Sa3int phage family, the bacteriophage ɸ13 shares features with the other members. These features include the Sa3 type integrase and the presence of the *sak*, *chp*, and *scn* virulence genes (Goerke et al., 2009; Xia & Wolz, 2014), but an analysis of the genetic switches within the group has never been reported.

To characterize the switch from phage ɸ13, we have compared its switch region to the switch regions in TP901‐1 and the switch regions in *Staphylococcus* phages, showing that a group of phages with the Sa3 integrase shares similar switch regions containing CI and MOR homologs. We have purified the ɸ13 CI repressor and shown that it interacts with three palindromic sites in the switch region. By constructing plasmids containing the ɸ13 genetic switch with *lacZ* reporter fusions to the lytic promoter (switch plasmids) and analysis of the frequency of Lac phenotypes in transformants of the natural and a heterologous host, we have shown that a functional *mor* gene is required for “decision switching” and that this process is independent of staphylococcal host factors. The concept of “toggle switching” has previously been defined as “the induced or accidental change from one stable phenotype to another from a bistable switch” (Gardner et al., 2000). Thus, toggle switching is when the phage changes its state, either spontaneously or following induction. In contrast, decision switching is when the phage initially establishes itself as lytic or lysogenic, in a process that precedes toggle switching. In the current study, ɸ13 switch plasmids were found to toggle spontaneously from the “lytic” (Lac+, blue) colony phenotype to a stable “lysogenic” (Lac−, white) colony phenotype. In the opposite direction, toggling from the stable “lysogenic” colony phenotype to the “lytic” colony phenotype could readily be induced by sub‐lethal concentrations of DNA damaging agents. The results from this study can prove important in the analysis of *S. aureus* virulence in vivo by determining conditions during infection that would result in induction and spread of ɸ13 and the unwanted establishment of ɸ13 prophages in susceptible hosts. The results may ultimately aid in the understanding of how some Sa3int prophages are disseminated in society and suggest ways to limit *S. aureus* colonization in humans.

## 2 EXPERIMENTAL PROCEDURES

### 2.1 Growth specifications

Unless otherwise specified, *E. coli* and *Bacillus subtilis* were grown in lysogeny broth (LB) or LB agar, while *S. aureus* was grown in tryptic soy broth (TSB) or agar (TSA). All strains were grown at 37°C with sufficient aeration and the addition of appropriate antibiotics. 5‐Bromo‐4‐chloro‐3‐indolyl‐β‐d‐galactopyranoside (X‐gal) was added to agar plates at 100–200 µg ml^−1^.

Phages were stored and diluted in SM buffer (100 mM NaCl, 8 mM MgSO_4_·7H_2_O, 50 mM Tris‐CL (pH 7.5)).

### 2.2 Bacterial strains and plasmids

All bacterial strains used in this study can be found in Table A1, while selected strains, plasmids, and phages are summarized in Tables 1, 2, 3, respectively.

**TABLE 1:** List of selected bacterial strains used in this study

Strain	Species	Short description or relevant genotype	Reference or source
168	*B. subtilis*		Burkholder and Giles (1947)
8325‐4	*S. aureus*	NCTC8325 phage cured	Novick (1967)
8325‐4 ɸ13‐kana	*S. aureus*	8325‐4 lysogenized with ɸ13‐kana	Tang et al. (2017)
CSK8	*B. subtilis*	168 *thrC*::pCSK2 expressing the “lytic” phenotype	This study
CSK9	*B. subtilis*	168 *thrC*::pCSK2 expressing the “lysogenic” phenotype	This study
CSK10	*B. subtilis*	168/pCSK3 expressing the “lytic” phenotype	This study
CSK11	*B. subtilis*	168/pCSK3 expressing the “lysogenic” phenotype	This study
CSK32	*S. aureus*	8325‐4/pCSK3 expressing the “lytic” phenotype	This study
CSK33	*S. aureus*	8325‐4/pCSK3 expressing the “lysogenic” phenotype	This study
CSK47	*S. aureus*	CSK45 *geh*::pCSK9 expressing the “lytic” phenotype	This study
CSK48	*S. aureus*	CSK45 *geh*::pCSK9 expressing the “lysogenic” phenotype	This study
CSK56	*S. aureus*	CSK46 *geh*::pCL25	This study
CSK59	*S. aureus*	8325‐4/pNZlac	This study
CSK68	*S. aureus*	CSK45 *geh*::pCL25	This study

Note

Strain, species, a short description, and reference of selected strains. See Table A1 for a complete list.

**TABLE 2:** Plasmids used in this study

Plasmid	Description	Reference or source
pDG1729	Promoterless LacZ reporter plasmid integrating in *B. subtilis* in *thrC*	Guérout‐Fleury et al. (1996)
pNZlac	Promoterless replicative LacZ reporter plasmid	Kovács et al. (2010)
pCSK2	ɸ13 switch inserted in pDG1729, *lacZ* fused to P_L_ downstream of *mor*	This study
pCSK3	ɸ13 switch inserted in pNZlac, *lacZ* fused to P_L_ downstream of *mor*	This study
pCL25	Promoterless integration plasmid integrating into *attB* ^L54a^ in *geh* of *S. aureus*	Luong and Lee (2007)
pYL112Δ19	Contains L54a integrase. Needed for integration of pCL25 derivatives in *S. aureus*	Lee et al. (1991)
pCSK9	ɸ13 switch and *lacZ* of pCSK2 inserted in pCL25	This study
pCSK11	As pCSK2, with disrupted *mor* start codon (ATG to ATC)	This study
pCSK12	As pCSK9, with disrupted *mor* start codon (ATG to ATC)	This study
pCSK13	As pCSK3, with disrupted *mor* start codon (ATG to ATC)	This study
pET30a(+)	Expression vector containing ɸ13 rCI gene	This study

Note

Plasmid name, a short description, and reference included for all plasmids.

**TABLE 3:** Phages used in this study

Phage	Description	Reference
ɸ13‐kana	Fully functional ɸ13 containing a kanamycin resistance cassette	Tang et al. (2017)
ɸ11	Generally transducing *S. aureus* phage	Novick (1967)
ɸ80α	Generally transducing *S. aureus* phage	Novick (1963)
SPP1	Generally transducing *B. subtilis* phage	Riva et al. (1968)

Note

Phages used, a brief description of these, and a reference of origin included.

### 2.3 Protein expression and purification

**FIGURE A1:**
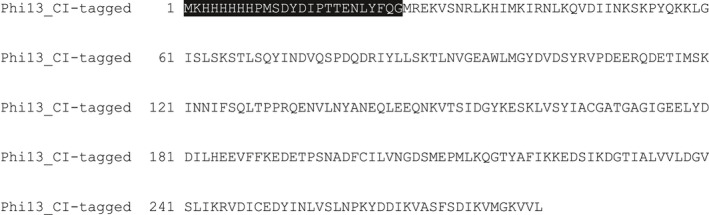
The protein sequence of ɸ13 rCI used in this study. The added tag is marked with a black box and consists of the His‐tag (HHHHHH) and a TEV cleavage site (ENLYFQG). Cleavage of the tag leaves the G

A synthetic codon‐optimized gene for recombinant CI repressor protein with an N‐terminal His‐tag and TEV cleavage site (ɸ13 rCI) was purchased from Genscript (Figure A1) in a pET30a(+) expression vector. The expression vector was transformed into BL21 (DE3) *E. coli* cells and grown at 37°C. ɸ13 rCI expression was induced by 0.5 mM isopropyl β‐d‐1‐thiogalactopyranoside in a mid‐exponential culture and the temperature lowered to 25°C.

After 20 h, cells were harvested and sonicated. The cell lysate was collected and filtered through a 0.22 μm filter. Chromatographic purification was done on an ÄKTA purifier 10 system (GE Healthcare Life Sciences) by immobilized metal affinity chromatography (IMAC; HisTrap HP 1 ml column) and size exclusion chromatography (SEC; HiLoad 26/600 Superdex200 column). 20 mM Tris, 20 mM imidazole, 1 M NaCl, pH 8.0 was used as washing buffer and 20 mM Tris, 500 mM imidazole, 100 mM NaCl, pH 8.0 as elution buffer in IMAC, while 20 mM Tris, 100 mM NaCl, pH 7.5 was used as SEC buffer. Blue Dextran (2000 kDa), Ferritin (440 kDa), Catalase (232 kDa), Aldolase (158 kDa), Carbonic Anhydrase (29 kDa), and Aprotinin (6.5 kDa) were used as Mw standards in SEC.

Protein purity was assessed by SDS‐PAGE (15% gels).

The protein concentration was estimated by A_280_ using an extinction coefficient of 26 360 M^−1^ cm^−1^ (tagged) and 23 380 M^−1^ cm^−1^ (untagged) as estimated with ProtParam (http://web.expasy.org/protparam) (Gasteiger et al., 2005).

For EMSA using native CI, the tag on ɸ13 rCI was removed beforehand by mixing the protein 10:1 with TEV protease (Sigma Aldrich) and incubating it at 4°C for 3 days. The uncleaved protein was separated from the mixture by IMAC and the flowthrough was collected for use.

### 2.4 DSF and CD spectroscopy

The inflection temperature, *T*_i_, was determined using a Nanotemper Tycho™ NT.6 DSF monitoring changes in the ratio of intrinsic fluorescence at 350 and 330 nm while heating from 35°C to 95°C with a temperature ramp of 30°C min^−1^. The sample was loaded in high precision glass capillaries with a concentration of 4.9 µM.

For CD spectroscopy, the purified protein was transferred to a 20 mM NaF buffer, pH 7.5. The experiments were conducted on a Jasco J‐815 CD spectropolarimeter in a suprasil quartz cell with a 1 mm light path (Hellma Analytics) and a sample concentration of 1.56 µM. The spectra were measured at room temperature in a continuous mode at 20 nm min^−1^ from 260 to 190 nm with a data pitch and bandwidth of 1 nm, averaging five spectra per measurement and subtracting the buffer spectra. The CD spectrum was deconvoluted and analyzed using DichroWeb (Whitmore & Wallace, 2008) with the CDSSTR method (Sreerama & Woody, 2000) and reference sets 3 and 6 and by BeStSel (Micsonai et al., 2015, 2018).

### 2.5 Synthesis and labeling of DNA fragments

For gel shift assays, DNA fragments containing each of the three operator sites, O_R_, O_L_, and O_D_, were synthesized by PCR with appropriate annealing temperatures using a Phusion polymerase. Non‐fluorescent probes were created by amplifying the genetic switch region of ɸ13 with primers MK867 and MK868 for O_R_, MK869 and MK870 for O_L_, and MK871 and MK872 for O_D_. The DNA fragments were made fluorescent by a second PCR run with the Cy5‐containing primers MK740 and MK741. Mutated probes with altered operator half‐sites (TTCA to TTGA) were amplified from the non‐fluorescent probes in two steps. First overlapping left and right half parts were amplified: O_R_ left part from the O_R_ probe using MK1038 and MK741; O_R_ right part from the O_R_ probe using MK1039 and MK740; O_L_ left part from the O_L_ probe using MK1040 and MK741; O_L_ right part from the O_L_ probe using MK1041 and MK740; O_D_ left part from the O_D_ probe using MK1042 and MK741; O_D_ right part from the O_D_ probe using MK1043 and MK740. Full‐length mutant probes were synthesized with the overlapping left parts and right parts as templates and mega‐primers in a subsequent PCR reaction, followed by amplification of the product with fluorescent primers MK740 and MK741. See Table A2 for a list of primers used in this study.

### 2.6 EMSA

Tagged and detagged ɸ13 rCI was mixed with binding buffer (100 mM Tris‐HCl, 5 mM EDTA, 500 mM NaCl, 5 mM DTT, 25% glycerol), bovine serum albumin, and sheared DNA. In each assay, ~0.5 nM DNA probe was mixed with varying concentrations of rCI in a total of 20 µl binding solution. After pre‐incubation on ice for 15 min, the DNA fragment was added and incubation was continued for 30 min. The mixture was then transferred into the empty wells of a chilled 2% agarose gel and run horizontally in 1× TBE (Tris/Borate/EDTA) buffer for 90 min at a constant voltage of 110 at 0°C. For visualization and quantification, the gel was scanned directly in a STORM 860 Imager (Amersham Biosciences) using the red channel (635 nm) at high sensitivity, followed by image editing using the ImageQuant TL software (Amersham Biosciences). The image was analyzed using Fiji (Schindelin et al., 2012). The integrated density of each band was measured and used to calculate the individual fractions of protein bound to DNA at each protein concentration. The data were fitted using the non‐linear curve fitting option and the specific binding with Hill slope option in GraphPad Prism (GraphPad Software, Inc.).

### 2.7 Construction of switch plasmids

All PCRs were conducted at appropriate annealing temperature and elongation time using Phusion polymerase unless otherwise specified. All switch plasmids contained the entire genetic switch of ɸ13 with a *lacZ* gene translationally fused to P_L_ downstream of *mor*, and all constructs were verified by sequencing. pCSK2 was created by amplifying the genetic switch region of ɸ13 from 8325‐4 ɸ13 with primers MK860 and MK863, digestion by EcoRI and BamHI, followed by ligation into digested pDG1729. From pCSK2, the switch region and *lacZ* gene were amplified by primers MK863 and MK902, digested by EcoRI and BglII, and ligated into pCL25 digested by EcoRI and BamHI creating pCSK9. pCSK3 was constructed by amplifying the switch region of ɸ13 from 8325‐4 ɸ13 with primers MK861 and MK862, digestion by XbaI and SphI, followed by ligation into digested pNZlac. The start codon of *mor* was mutated (ATG to ATC) in pCSK2, pCSK3, and pCSK9 by use of CloneAmp™ HiFi PCR Premix Polymerase resulting in pCSK11, pCSK13, and pCSK12. The primers MK914 and MK915 were used to amplify the entire plasmid while incorporating the point mutation.

### 2.8 Transformations

Electrocompetent *E. coli* cells were created and electroporated as previously described (Pedersen et al., 2020). Cells were plated on selective agar plates after 1 h of recovery.

Competent *B. subtilis* cells were obtained as described in (Konkol et al., 2013) with one minor change; 30 min after plasmid addition equal volumes of LB media were added to the transformation mixture, followed by incubation for 1 h and plating on selective plates.

Transformations in *S. aureus* were performed using the procedure for electroporation described in (Monk et al., 2015). pYL112Δ19 was transformed into 8325‐4 and 8325‐4 ɸ13‐kana before the introduction of integrating plasmids to supply the required integrase.

### 2.9 Transductions

Transductions in *B. subtilis* and *S. aureus* were accomplished by using phage SPP1 or ɸ11/ɸ80α, respectively. See Table 3 for all phages used in this study. Transducing phage stocks were prepared as plate lysates on the donor strains by allowing phage adsorption for 15 min at 37°C with 5 mM CaCl_2_ and subsequent plating in top agar (LB/TSB, 0.5% agar, 5 mM CaCl_2_). After overnight incubation, the top layer was suspended in SM, centrifuged at 5000 *g* for 10 min, and the supernatant was filter‐sterilized (0.2 µm). Transducing phages were added at a multiplicity of infection (MOI) of 10 to *B. subtilis* 168 and MOI = 0.1 to *S. aureus* 8325‐4 and 8325‐4 ɸ13, followed by 15 min incubation at 37°C. Cells were washed with equal volumes of 20 mM Na‐citrate and plated on selective plates containing X‐gal and 20 mM Na‐Citrate.

### 2.10 Stability of switch plasmid phenotypes

To examine the stability of the “lysogenic” and “lytic” phenotype of the switch plasmids, a single colony resulting from transformation was suspended in an appropriate medium, diluted, and plated at a density allowing the formation of single colonies for phenotype determination.

### 2.11 Plate inductions

To test the effect of inducing substances, both *B. subtilis* and *S. aureus* cells containing switch plasmids were plated at a cell density resulting in around 1000 colonies per plate, obtained by suspending a colony in LB/TSA medium, dilutions, and plating; 3 µl inducing substance (0.5 mg ml^−1^ mitomycin C, 1 mg ml^−1^ ciprofloxacin, or 30% hydrogen peroxide) was applied to the middle of the plate, followed by incubation at 37°C. When relevant, selected colonies were suspended in media, diluted, and plated at a density resulting in single colonies to determine phenotypes.

### 2.12 Temporal induction

A culture of 8325‐4 *geh*::pCSK9 favoring the “lysogenic” phenotype (CSK48) was inoculated in TSB and grown to OD_600_ = 0.6 followed by induction by 1 µg ml^−1^ mitomycin C. Samples were taken periodically, diluted in TSB, and plated on selective TSA plates containing X‐gal. Switch plasmid phenotypes were determined after overnight incubation.

### 2.13 Phage infection of 8325‐4 harboring switch plasmids

Cultures were inoculated in TSB and grown to OD_600_ = 0.6. At this point, a sample was plated for determination of colony forming units (CFU) ml^−1^, after which, phages were added at a MOI of 0.1, followed by incubation for 2 h. Cultures were spun at 5000 *g* for 5 min and the supernatant filter‐sterilized (0.2 µm), followed by dilution in SM buffer, titration on bacterial lawns of 8325‐4 (TSB, 0.5% agarose, 5 mM CaCl_2_), and overnight incubation for determination of plaque forming units (PFU) ml^−1^. The amount of phages produced per host cell was determined by calculating PFU ml^−1^ per CFU ml^−1^.

### 2.14 Lysogenization frequency of ɸ13

Exponential cultures of 8435‐4 were infected with ɸ13‐kana at a MOI of 0.1 and 1. Phages were allowed to adsorb for 15 min on ice, after which cells were centrifuged and the pellet was resuspended in SM buffer. Dilutions were plated on TSA plates containing kanamycin for the determination of lysogens. Dilutions were further titrated on bacterial lawns of 8435‐4 to detect cells in which the phage favored the lytic lifecycle. Phage titer was determined as PFU ml^−1^ after overnight incubation. The lysogenization frequency was calculated as the ratio of lysogenic cells (CFU (Kan^R^) ml^−1^) of all infected cells (CFU (Kan^R^) ml^−1^ + PFU ml^−1^).

## 3 RESULTS AND DISCUSSION

### 3.1 Topology of the ɸ13 switch region and detection of identical switches in members of the Sa3int and the PVL encoding phages

**FIGURE 1:**
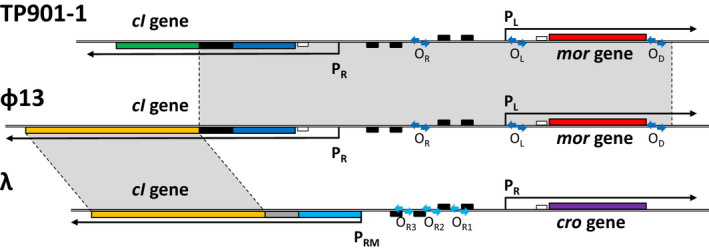
Comparison of switch regions from *S. aureus* phage ɸ13, *L. lactis* phage TP901‐1, and *E. coli* phage λ. The geometry of the switch region is not shown to scale. The *mor* gene of TP901‐1 and ɸ13 is shown as a red box, the *cro* gene of λ is shown as a purple box, and the first part of the *cI* gene encoding the DNA binding N‐terminal domain (CI‐NTD) is shown as a blue box. The part of the *cI* gene from ɸ13 encoding the extreme C‐terminal domain (CI‐CTD_2_) is not homologous to the CI‐CTD_2_ domain in TP901‐1 but to the C‐terminal domain from CI of λ, containing a peptidase domain. CI‐CTD_2_ is shown as a green box for TP901‐1 and a yellow box for ɸ13 and λ. A dimerization domain (CI‐CTD_1_) with helical hook structures in TP901‐1 connects CI‐CTD_2_ and CI‐NTD and is shown as a black box for TP901‐1 and ɸ13, while the dimerization region is shown as a gray box for λ. The divergently oriented promoters, P_R_ and P_L_ for TP901‐1, P_RM_ and P_R_ for λ, and putative P_R_ and P_L_ promoters for ɸ13, are shown as arrows preceded by small black boxes symbolizing −10 and −35 regions. Small white boxes indicate ribosomal binding sites. Operators O_R_, O_L_, and O_D_, recognized by the CI‐NTD in TP901‐1, homologous regions for ɸ13, and operators O_R1_, O_R2_, and O_R3_ for λ, are shown as blue arrows. Figure inspired by Pedersen et al. (2020)

**FIGURE A2:**
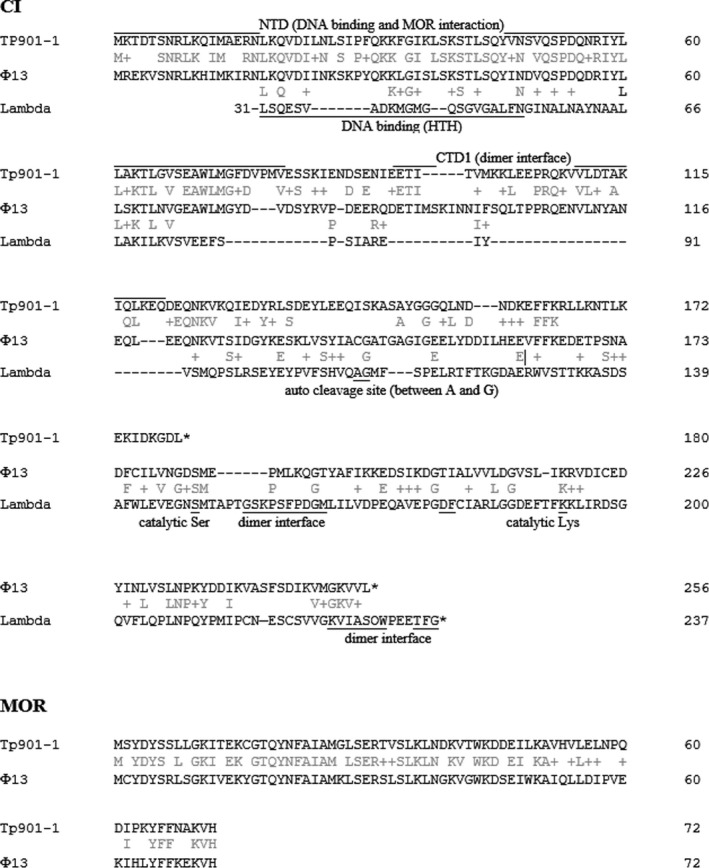
Sequence alignments of CI from *L. lactis* phage TP901‐1, *S. aureus* phage ɸ13, and *E. coli* phage λ (top) and MOR from TP901‐1 and ɸ13 (bottom). Identical and similar residues are shown in gray. Known domains are indicated by lines and designated by brief descriptions. The overall similarity between the CI repressors from ɸ13 and TP901‐1 is 51% identical residues, while the local domain similarity for the NTD and CTD_1_ is 71% and 39%, respectively. The overall similarity between CI from ɸ13 and λ is 19% identical residues, where the N‐terminal parts only show similarities in the DNA binding domains. In the C‐terminal part, where λ carries its peptidase domain required for autocleavage, the local similarity is 21%, but the catalytic Ser and Lys residues and the G‐residue of the autocleavage site are conserved. The overall similarity for the MOR anti‐repressors from ɸ13 and TP901‐1 is 65%

Recently, a global search for DNA sequences similar to the phage TP901‐1 switch region led us to identify a putative switch region in the *S. aureus* phage ɸ13 (Pedersen et al., 2020). As shown in the map in Figure 1 and the amino acid sequence alignments in Figure A2, the homology spans the N‐terminal part of the *cI* gene, the intergenic region, and a divergently oriented, putative *mor* gene. Putative P_L_ and P_R_ promoters and O_R_, O_L_, and O_D_ operators were recognized in the ɸ13 switch in similar locations as in TP901‐1 (Pedersen et al., 2020). The limited similarity was found to the C‐terminal domain CI‐CTD_1_ from TP901‐1, whereas the CI‐CTD_2_ showed no sign of conservation (Figure A2). Instead of the CI‐CTD_2_ domain, the CI repressor from ɸ13 carries a region with clear similarity to the C‐terminal domain of the CI repressor from phage λ (see Figure A2). The two catalytic residues from the peptidase domain as well as the autocatalytic site from λ are conserved in ɸ13, suggesting that the CI repressor from ɸ13, in contrast to the CI repressor from TP901‐1 (Madsen et al., 1999), could be capable of autocleavage.

To analyze if the ɸ13 switch type was widespread among phages, we performed a homology search (Altschul et al., 1990) with the 1260 bp ɸ13 DNA sequence covering the region shown in Figure 1, excluding staphylococcal genome sequences. Only sequences from completed genomes of staphylococcal phages were identified, and six phages had hits with full coverage and sequence identity (>99% identity): PVL, tp301‐1, P630, 3AJ‐2017, IME1346_01, and ɸ13. Two of these, 3AJ‐2017 and IME1346_01, encode the virulence proteins Sac, Chp, and Scn (Oliveira et al., 2019) and belong to the Sa3int group like ɸ13, while the phages PVL and tp301‐1 encoded the LukS‐PV and LukF‐PV PVL virulence factors (Oliveira et al., 2019). This shows that the homology between the switches from ɸ13 and TP901‐1 is not unique and that the ɸ13 switch type determines the decisions between lysogenic and lytic growth for two important groups of phages carrying pathogenicity genes.

### 3.2 ɸ13 CI binds to operator sites in the intergenic region between *cI* and *mor*

**FIGURE 2:**
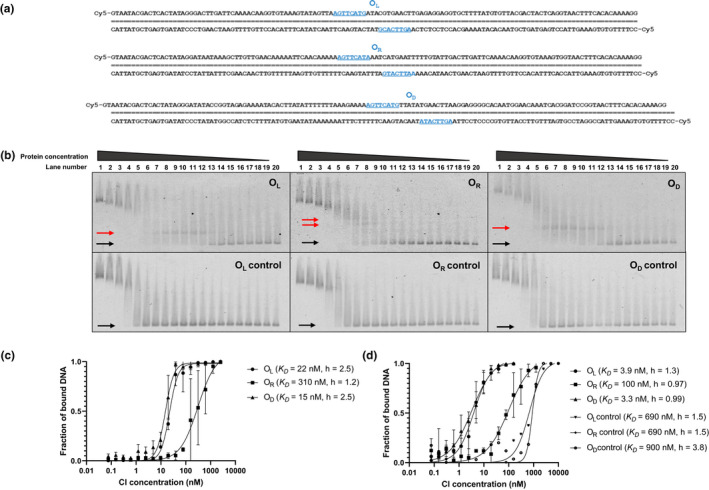
EMSA of rCI repressor binding to putative operators O_R_, O_L_, and O_D_ in the ɸ13 switch. (a) Nucleotide sequence of the DNA fragments carrying ɸ13 O_L_, O_R_, and O_D_. Operator sites are underlined in blue. (b) Fluorescent scan of agarose gels from EMSA employing ɸ13 rCI and DNA fragments carrying O_L_, O_R_, and O_D_ and of agarose gels from EMSA employing ɸ13 rCI and DNA fragments carrying mutated operator sites, in which the central C in each half‐operator site was exchanged for a G: O_R_^control^, O_L_^control^, and O_D_^control^. Lane numbers are listed, where the concentration of rCI in lane 1 is 16.9 µM and in the following lanes diluted serially by two‐fold dilutions. Lane 20 contains free unbound DNA. Black arrows point at bands with unbound DNA probe and red arrows point at bands for CI‐bound DNA probe. Smears at high CI concentrations are presumed to be CI complexes bound to DNA probe. (c) Analysis of the degree of binding (CI‐bound DNA/(unbound DNA + CI‐bound DNA) from quantification of the bands for unbound DNA probe (black arrows in “b”) and the bands for CI‐bound DNA probe (red arrows in “b”), using the non‐linear curve fitting option and the specific binding with Hill slope option in GraphPad Prism. Binding curves, dissociation constants, and Hill coefficients from the best fits are shown. Triplicate biological replicas were analyzed for the determination of specific binding and standard deviations from these are shown. (d) Analysis of the degree of binding calculated as (DNA in lane 20 – free DNA)/DNA in lane 20) from quantification of only the bands for unbound DNA probe (black arrows in b). Binding curves, dissociation constants, and Hill coefficients from the best fits are shown. Triplicate biological replica was analyzed for the determination of specific binding, and error bars represent ±one standard deviation

**FIGURE A3:**
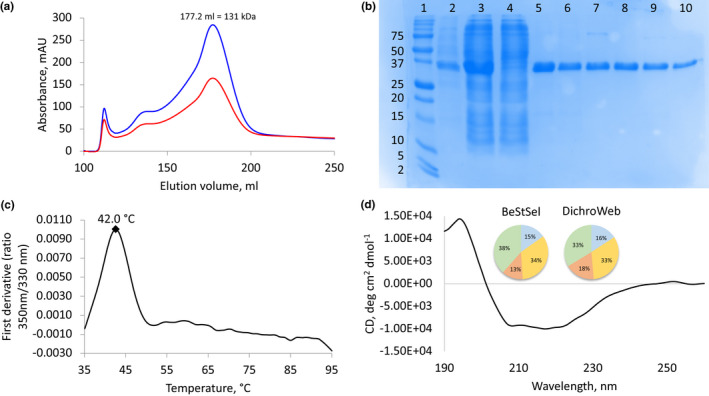
Purification and characterization of tagged ɸ13 rCI. (a) SEC chromatogram of ɸ13 rCI. The absorbance at 260 and 280 nm are shown in red and blue, respectively. The largest peak eluted at 177.2 ml corresponding to a tetramer. (b) SDS‐PAGE gel showing the different steps of expression and purification of ɸ13 rCI. Lane 1: Precision Plus Protein™ Dual Xtra Prestained Protein Standard marker, lane 2: cell pellet diluted in water, lane 3: cell lysate after sonication and centrifugation, lane 4: HisTrap HP 1 ml flowthrough, lane 5: HisTrap HP 1 ml eluate, lane 6–10: five fractions from SEC covering the peak from 110 to 185 ml. The molecular weights of standards in kDa are shown on the left. (c) The first derivative of the ratio between 350 and 330 nm measured with DSF. The point of the inflection temperature is marked with a diamond. (d) CD spectrum of ɸ13 rCI with the results from the deconvolution using BeStSel and DichroWeb inserted as pie charts. The predicted secondary structure types of helix, strand, turns, and unordered are colored blue, yellow, red, and green, respectively, for both BeStSel and DichroWeb predictions

To characterize the switch present in ɸ13, it was first important to know that the postulated CI repressor was functional and had an affinity towards the postulated CI operators. To determine whether the CI repressor from ɸ13 binds to these putative operators, CI was expressed with an N‐terminal His‐TEV‐tag (termed rCI, see Figure A1) and purified by affinity chromatography and gel filtration (Figure A3a,b). After circular dichroism spectroscopy and differential scanning fluorimetry had confirmed that the protein was folded and stable (Figure A3c,d), electrophoretic mobility shift assays (EMSA) were performed with this protein preparation using fluorescent probes carrying O_R_, O_L_, or O_D_ from ɸ13 (Figure 2a). Agarose gel electrophoresis of binding reactions with increasing CI concentration followed by detection of the labeled DNA by fluorescence scanning showed that the rCI protein bound the O_L_ and O_D_ sites with high affinity (dissociation constants of 22 and 15 nM, respectively), but with lower affinity to the O_R_ site (dissociation constant of 310 nM) as shown in Figure 2b,c. While the operator half‐sites of the O_L_ and O_D_ sequences correlated with the TP901‐1 consensus (AGTTCAYR), one of the O_R_ half‐sites deviated by one base (A **A** TTCATA). This difference could potentially explain the higher dissociation constant for binding to O_R_. To validate that the putative binding sites were responsible for the binding to rCI, a series of mutated fluorescent DNA probes were produced, which were identical to the probes shown in Figure 2a, except that the central C nucleotide was mutated to G. Binding of rCI to these mutated control probes was severely weakened and no bands for specific binding could be detected (Figure 2b).

Interestingly, it can be seen from Figure 2b, that the bands with CI‐bound DNA probe (marked with red arrows) disappear at higher concentrations of CI. We hypothesize that this is due to multimerization of CI because the labeled DNA probe migrates slowly as a smeared band at very high CI concentrations (Lane 1 in all gels in Figure 2b). To evaluate if the secondary binding events affected the determination of the dissociations constants, we plotted the missing fraction of the free DNA probe (i.e., the fraction of DNA probe that was retarded by any means) against the CI‐concentration. Using this quantification method, the apparent dissociation constants for CI binding to the O_L_, O_D_, and O_R_ probes were found to be 3.9, 3.3, and 100 nM, respectively (Figure 2d), showing that the secondary binding events affected the determination of the dissociation constants. Also, the Hill coefficients for O_L_ and O_D_ binding were changed from 2.5 to 1.3 and 2.5 to 1.0, respectively (Figure 2c). The quantification of only the free DNA probe enabled the determination of apparent dissociation constants for binding of CI to the negative controls. Dissociation constants were detected at ~690, 900, and 690 nM, for the O_L_^control^, O_D_^control^, and O_R_^control^ probes, respectively, ten‐fold and hundred‐fold higher than the dissociation constants for the unmutated probes. In combination, these results show that the CI repressor from ɸ13 is functional and that the proposed operators are indeed binding sites for the repressor. The dissociation constants are somewhat lower than for binding of TP901‐1 CI to DNA containing TP901‐1 operators, found by mobility shift assays (Johansen et al., 2003; Pedersen & Hammer, 2008), but recent mobility shift assays of TP901‐1 CI binding to O_L_ using the same technique employed in this study quantified the dissociation constant to 2.9 nM (Pedersen et al., 2020). It is important to mention that the binding of CI to O_R_ has introduced some interesting challenges. Preliminary analysis of operator binding to the O_L_, O_D_, and O_R_ operators using purified CI, in which the His‐TEV‐tag had been cleaved off, showed a dissociation constant that was slightly higher than shown above for binding to probes containing the O_L_ and O_D_ operators (~12 nM). Of more importance, the binding of the O_R_ probe to this CI fraction showed dissociation constants equaling the O_L_ and O_D_ operators (12 nM). This issue needs to be addressed in a future study, but the results obtained here and presented in Figure 2 have established that the proposed O_L_, O_D_, and O_R_ operators are specific binding sites for the ɸ13 CI repressor.

### 3.3 A minimal 1.3 kb switch region from ɸ13 is capable of decision switching in *S. aureus*

**FIGURE 3:**
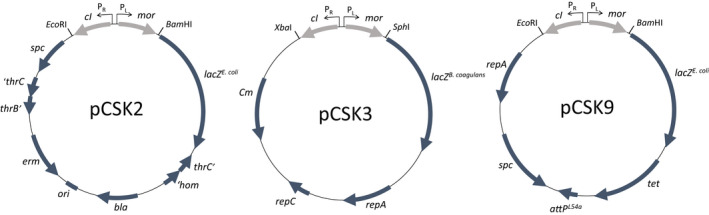
Reporter plasmids for monitoring switching of the ɸ13 switch in *B. subtilis* and *S. aureus*. The genetic switch of ɸ13 was inserted into the reporter plasmids pDG1729 (pCSK2), pNZlac (pCSK3), and pCL25 (pCSK9), allowing expression of the *lacZ* reporter gene at P_L_ activity. pCSK3 is capable of replication in *B. subtilis*, while pCSK2 lacks a replicon and needs to integrate by homologous recombination into the *thrC* gene of *B. subtilis*. pCSK9 integrates into the *attB* ^L54a^ integration site in *S. aureus*, facilitated by a L54a integrase supplied by pYL112Δ19. Plasmid maps are not to scale. Restriction sites used for insertion of the switch DNA are shown. *cI* and *mor* of the ɸ13 genetic switch are illustrated in a lighter gray. Other contained genes: *spc*: spectinomycin resistance not expressed in *S. aureus*, *lacZ^E. coli^*: beta‐galactosidase gene originating from *E. coli* fused to the ribosome binding site of *B. subtilis spoVG*, ‘*hom*, *thrC*’, ‘*thrC*, and *thrB*’: segments of the genes flanking the integration site of pCSK2 in *B. subtilis* allowing integration by homologous recombination, *LacZ^B. coagulans^*: beta‐galactosidase gene originating from *B*. *coagulans*, *repA* and *repC*: replicon A and C, *Cm*: chloramphenicol resistance gene, *tet*: tetracycline resistance, *attP^L54a^*: L54a phage attachment region

After having shown that the *cI* encoded repressor can bind to an operator (O_L_) overlapping the putative P_L_ promoter as in the TP901‐1 switch, the isolation of a functional ɸ13 switch for in vivo analysis of switch frequencies appeared to be feasible. Previously a DNA fragment spanning the *cI* and *mor* genes from TP901‐1 had successfully been inserted into a promoter fusion plasmid (Madsen et al., 1999), and used for detailed characterization of the TP901‐1 switch (Pedersen & Hammer, 2008) through transformation of its *L. lactis* host. To monitor the regulatory decisions upon introduction of ɸ13 switch DNA, we constructed plasmids with transcriptional fusions to the P_L_ promoter of the ɸ13 switch region, as illustrated in Figure 3, with a *lacZ* reporter gene located after the *mor* gene. Upon transformation and plating on selective plates containing X‐gal, the resulting colonies were either blue, indicative of high *mor* expression from the lytic P_L_ promoter, referred to as the “lytic” phenotype, or white, indicative of low *mor* expression due to repression of the P_L_ promoter by CI expressed from the lysogenic P_R_ promoter, referred to as the “lysogenic” phenotype.

Plasmid pCSK3 replicates in *S. aureus* and upon transformation of strain 8325‐4 with pCSK3 we observed an average of 4.8% transformants with the “lysogenic” colony phenotype and 95% with the “lytic” phenotype (Table 4). This result indicated that the switch region present in pCSK3 could indeed perform decision switching in *S. aureus*. The genetic integrity of the switch region was verified by purification and subsequent re‐transformation of pCSK3 isolated from 8325‐4/pCSK3 transformants, yielding similar decision frequencies (see footnote to Table 4). Since the decision analysis was performed using a plasmid and not an intact phage, the use of pCSK3 as a model system for the ɸ13 decision switching required that the frequency of the “lysogenic” phenotype reflected the true lysogenization frequency. Infection of strain 8325‐4 with the ɸ13‐kana phage was therefore performed at a MOI of 1 and 0.1 at a bacterial concentration of 1.3 × 10^8^ CFU ml^−1^. As shown in Table A3, the average lysogenization frequency from five replicates was 4.6% ± 2.8% and 3.5% ± 3.3% for infections at an MOI of 1, and 0.1, respectively. Since the pCSK3 “lysogenic” (4.9%) and ɸ13 lysogenic frequencies are not significantly different (*p* = 0.87 and 0.47, for the two MOIs, respectively), we believe that the pCSK3 switch plasmid is a faithful biological model for ɸ13 switching. Finally, to ensure the importance of the MOR protein for the lytic pathway of the ɸ13 decision process, the start‐codon in the *mor* gene was changed to a stop codon. The resulting plasmids, pCSK12, and pCSK13 acquired the “lysogenic” phenotype in 100% of transformations when introduced into *S. aureus* 8325‐4, showing that the “lytic” phenotype is dependent upon the MOR protein (Table A4).

**TABLE 4:** Frequencies of colonies with “lysogenic” phenotype after the introduction of ɸ13 switch plasmids into *S. aureus* or *B. subtilis* by transformation

*S. aureus* 8325‐4		*S. aureus* 8325‐4 ɸ13‐kana		*B. subtilis* 168	
pCSK3^a^	pCSK9^b^	pCSK3^c^	pCSK9^b^	pCSK3	pCSK2
4.9% ± 2.9% (*n* = 6)	12% ± 10% (77 colonies in total, *n* = 5)	99.6% ± 0.5% (*n* = 4)	100% ± 0% (63 colonies in total, *n* = 9)	3.8% ± 1.6% (*n* = 10)	3.0% ± 1.6% (*n* = 18)

Note

Switch plasmids were replicative (pCSK3) or required integration into the host (pCSK9 in *S. aureus*, pCSK2 in *B. subtilis*). Recipient bacteria were naïve (*S. aureus* 8325‐4 or *B. subtilis* 168) or ɸ13 lysogens (*S. aureus* 8325‐4 ɸ13‐kana). The number of biological replicates with more than 100 resulting colonies is stated in brackets. Frequencies of “lysogenic” transformant colonies on plates containing X‐gal were calculated as the frequency between “lytic” and “lysogenic” in each transformation event and the average is shown here. Standard deviations are based on differences between each event.

^a^ Re‐transformation of 8325‐4 by pCSK3 plasmids extracted from “lytic” and “lysogenic” colonies of 8325‐4/pCSK3 yielded frequencies of “lysogenic” transformant colonies of 11% and 5%, respectively.

^b^ Indicates <100 transformants obtained in each experiment. The number of “lytic” and “lysogenic” colonies obtained in total for all replicas followed by the total count of biological replicates (n) for each event are stated in brackets.

^c^ Re‐transformation of 8325‐4 by pCSK3 plasmids extracted from “lytic” and “lysogenic” colonies of 8325‐4 ɸ13‐kana/pCSK3 yielded frequencies of “lysogenic” transformant colonies of 3% and 11%, respectively. When these plasmids were used to transform 8325‐4 ɸ13‐kana, 100% “lysogenic” transformants were obtained.

To analyze whether the decision frequency of the ɸ13 switch was dependent upon replication of the plasmid containing the switch DNA during the decision process, we introduced the switch on an integrative plasmid, pCSK9. Plasmid integration occurs at the bacterial attachment site of the phage L54a located in the lipase gene *geh* of *S. aureus* and is catalyzed by the phage integrase provided by plasmid pYL112‐19 (Luong & Lee, 2007). Transformation of *S. aureus* 8325‐4 with pCSK9 yielded a higher number of “lysogenic” colonies than with pCSK3 (12% compared to 4.9%, see Table 4). However, the very low efficiency of transformation (in total 77 transformants from 5 individual transformations) resulted in a high degree of variation, preventing a reliable determination of the decision frequency. Despite this, the transformations permitted the isolation of pCSK9 transformants with both the “lysogenic” (CSK48) and “lytic” (CSK47) colony phenotype.

### 3.4 The “lysogenic” colony phenotype from switch plasmids confers ɸ13 immunity

To assess whether the “lysogenic” colony phenotype expressed from the switch plasmids was functionally equivalent with the immunity derived from the ɸ13 prophages, we first tested if a ɸ13 lysogen could influence the decision frequency of the switch plasmids. To this end, pCSK3 and pCSK9 were introduced by transformation into (immune) *S. aureus* carrying a fully functional derivative of ɸ13 containing a kanamycin resistance gene (8325‐4 ɸ13‐kana) integrated into the chromosome (Tang et al., 2017). Despite a small number of successful transformation events (63 transformants from 9 individual transformations), which prevented reliable quantification of the switching frequency, it was striking that we exclusively observed “lysogenic” transformants when the ɸ13‐kana lysogen was transformed with pCSK9 (Table 4, column 4), suggesting that the production of CI by the ɸ13 lysogen had influenced the decision process by pCSK9. Accordingly, the vast majority (99.6%) of pCSK3 transformants of *S. aureus* 8425‐4 ɸ13‐kana were “lysogenic” on X‐gal‐containing plates, showing a strong influence of lysogenic levels of CI on the decision by pCSK3. Interestingly, a small fraction of 0.4% established the “lytic” phenotype despite the presence of the ɸ13 prophage, verified by PCRs and kanamycin resistance. To analyze whether the switch or the reporter gene had mutated, plasmids were purified from the colonies that had escaped the immunity phenotype. Transformation of these plasmids into 8325‐4 with and without the ɸ13 prophage yielded decision frequencies similar to the original transformations (see footnote to Table 4), showing that the “lytic” colony phenotype was not a result of mutations.

**FIGURE 4:**
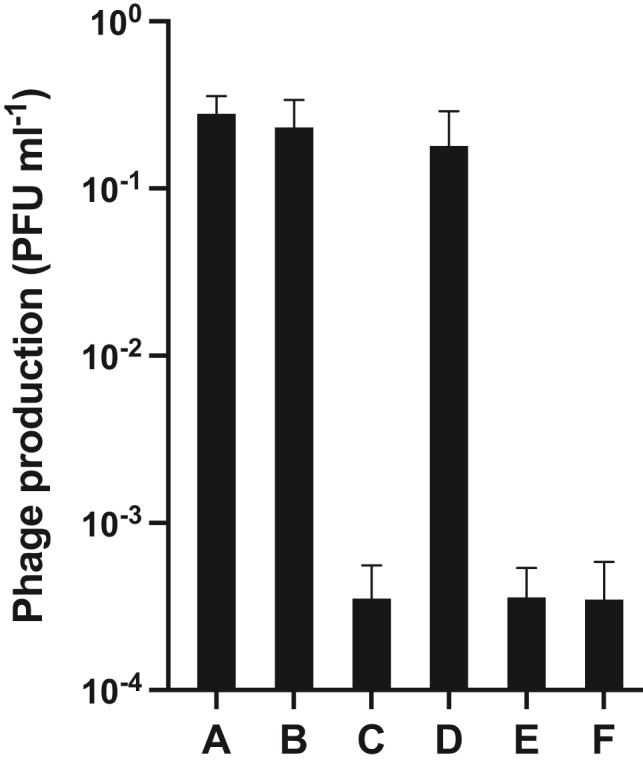
ɸ13‐kana infection of *S. aureus* 8325‐4 transformants carrying switch plasmids. Bacterial strains (A) 8325‐4 *geh*::pCL25 (CSK68), (B) 8325‐4/pNZlac (CSK59), (C) “lysogenic” 8325‐4 *geh*::pCSK9 (CSK48), (D) “lytic” 8325‐4 *geh*::pCSK9 (CSK47), (E) “lysogenic” 8325‐4/pCSK3 (CSK33), and (F) “lytic” 8325‐4/pCSK3 (CSK32) were infected by bacteriophage ɸ13‐kana at a MOI of 0.1. Phage production was quantified by normalizing the number of phages (determined as PFU ml^−1^ of the culture supernatant 2 h after infection) by the CFU ml^−1^ of the bacterial culture before infection. (a) and (b) are controls where the strains contain the empty vectors used for the construction of pCSK9 and pCSK3, respectively. Bars represent the mean value of three replicate experiments, while error bars represent standard deviations

We then investigated whether the plasmid‐derived “lysogenic” phenotype could confer ɸ13 immunity upon infection (Figure 4). When a liquid culture of the “lysogenic” 8325‐4/pCSK3 (CSK33) was infected with ɸ13‐kana, the “lysogenic” phenotype was found to restrict the production of phages (compare “B” and “E” in Figure 4). The same result was found when a transformant carrying the integrated pCSK9 plasmid (8325‐4 *geh*::pCSK9, CSK48) was inoculated from a “lysogenic” colony and was infected with ɸ13‐kana (compare “A” and “C” in Figure 4), showing that CI expressed from a chromosomally integrated ɸ13 switch conferred immunity to attacking ɸ13 phages. When cells with the integrated switch plasmid were inoculated from a “lytic” colony and infected with ɸ13, the presence of the switch did not affect phage production (compare “A” and “D” in Figure 4). Surprisingly, however, when cells containing the replicative plasmid pCSK3 were inoculated from a “lytic” colony and infected by ɸ13‐kana, the “lytic” switch phenotype inhibited phage production (compare “B” and “F” in Figure 4). It has previously been shown that replicating switch plasmids of TP901‐1 favoring P_L_ (“lytic” phenotype) resulted in partial immunity towards infecting phages (Madsen et al., 1999), in accordance with the present results. This suggests that, although there is not enough CI repressor present to repress P_L_ on the multi‐copy switch plasmid, the concentration is still sufficient to prevent lytic phage development. Upon screening of kanamycin resistance in cells surviving ɸ13‐kana infection, very few lysogenic cells were detected, showing that survival under these conditions is not due to the formation of ɸ13‐kana lysogens. Thus, the multi‐copy plasmid‐derived “lytic” switch phenotype appears to inactivate phage development in either direction.

### 3.5 Decision switching by the minimal switch region from ɸ13 does not require *S. aureus* host factors

As *E. coli* host factors are known to affect the integration and induction of phage λ (Roucourt & Lavigne, 2009), it was of interest to establish whether the ɸ13 switch can function independently of host factors. Switch plasmid pCSK3 has an origin of replication that is functional in most Gram‐positive bacteria (Kovács et al., 2010), and we could therefore also examine ɸ13 switching in *B. subtilis*. As *B. subtilis* is a heterologous host, it carries no specific staphylococcal factors but contains general Gram‐positive host factors. Upon transformation of pCSK3 into *B. subtilis* 168, we observed an average frequency of “lysogenic” colonies of 2%–3% (Table 4), which is close to the frequency obtained in *S. aureus*. This suggested that the decision frequency was not affected by specific host factors.

### 3.6 Low spontaneous induction frequency from the “lysogenic” phenotype to the “lytic” phenotype

To examine the stability of the ɸ13 “lysogenic” colony switch phenotype, simulating the stability of ɸ13 lysogenic cells, we suspended and plated a large number of cells from “lysogenic” colonies on agar plates containing X‐gal. Around 0.2% “lytic” colonies were detected in otherwise “lysogenic” colonies from both *B. subtilis* and *S. aureus*. This low level of toggling from the “lysogenic” to the “lytic” colony phenotype is hypothesized to be due to spontaneous induction of the SOS response, which is inevitable and leads to prophage induction in bacteria that contain an SOS response and an intact *recA* gene (Goerke et al., 2006). To distinguish the process of toggle switching from a mutational event leading to the “lytic” colony phenotype, we purified pCSK3 plasmid from “lytic” spontaneous revertants and re‐transformed them into the same host, with and without the ɸ13 prophage. After transformation of the strains with the extracted pCSK3 plasmid, we obtained a frequency of “lysogenic” transformant colonies at 5% for re‐transformation of 8325‐4, and at 100% for re‐transformation 8325‐4 ɸ13 kana, indistinguishable from frequencies obtained by transformation with the original plasmid, and showing that the change was epigenetic rather than mutational.

### 3.7 High toggle frequency of the ɸ13 switch from the “lytic” to the “lysogenic” phenotype

While the “lysogenic” colony phenotype was very stable, the “lytic” colony phenotype was subject to frequent toggling to the “lysogenic” phenotype. When the cells from small (young) “lytic” colonies were suspended in medium and plated on agar plates containing X‐gal, around 3% of the colonies had assumed the “lysogenic” phenotype. Plating of larger (older) “lytic” colonies resulted in an average of 60% “lysogenic” colonies. Because of the somewhat artificial nature of the “lytic” phenotype, the stability of the phenotype was not analyzed further.

### 3.8 Induction of the “lytic” switch phenotype by DNA damaging agents

**FIGURE 5:**
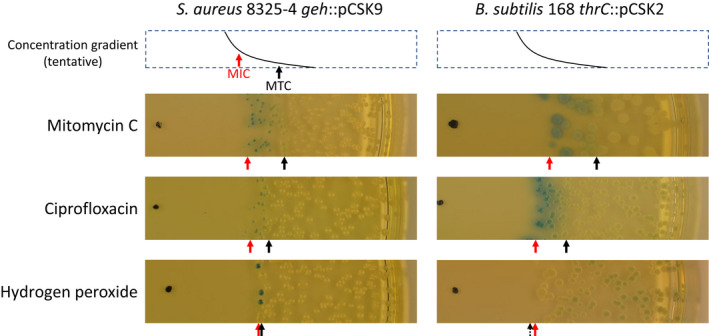
Induction of “lysogenic” transformants of *B. subtilis* 168 and *S. aureus* 8325‐4 by mitomycin C, ciprofloxacin, and hydrogen peroxide. Bacteria from a “lysogenic” (white) colony of *S. aureus* 8325‐4 *geh*::pCSK9 (CSK48, left) and *B. subtilis* 168 *thrC*::pCSK2 (CSK9, right) were suspended in media and plated on X‐gal plates. After drying, a drop of mitomycin C (top), ciprofloxacin (middle), or hydrogen peroxide (bottom) was applied in the middle. The plate was incubated overnight at 37°C. Estimated MIC and MTC values are shown as red and black arrows, respectively. The MTC value of *B. subtilis* 168 *thrC*::pCSK2 exposed to hydrogen peroxide cannot be determined, but an estimate is suggested by a dashed arrow

Above, we reported that 0.2% of cells that had grown in “lysogenic” colonies on an agar plate had spontaneously toggled to the “lytic” phenotype, possibly initiated by the spontaneous induction of the SOS response. A more dramatic visualization of the interplay between the DNA damage and the ɸ13 switch could be observed by exposing “lysogenic” cells to a gradient of DNA damaging agents on agar plates containing X‐gal. Induction of “lysogenic” (white) to “lytic” (blue) toggle switching is intimately connected to the level of the DNA damaging compound as seen in Figure 5. In our working hypothesis, the cellular CI concentration is determined through a race between CI expression and CI degradation, where a threshold CI concentration has to be crossed if the switch should toggle from the “lysogenic” to the “lytic” phenotype. Two clear zones can be detected among the bacterial colonies on the X‐gal‐containing plates at different distances from the DNA damaging agents: a death zone and a toggle‐inducing zone. The minimal inhibitory concentration (MIC) of the DNA damaging agent must be located at the border between growth and non‐growth. Accordingly, a minimal toggle‐inducing concentration (MTC) can be located at the border between purely white colonies and colonies with a bluish tint. For this analysis, a suspension of bacteria with the “lysogenic” (white) phenotype was plated on agar plates, and a drop of DNA damaging agent was applied to the center. Mitomycin C, ciprofloxacin, and hydrogen peroxide are all known to induce ɸ13 lysogens (Goerke, Koller, et al., 2006; Tang et al., 2017), and Figure 5 shows the response of the ɸ13 switch plasmid to gradients of DNA damaging agents in its natural host (Figure 5, left panel, 8325‐4 *geh*::pCSK9 (CSK48)) and in *B. subtilis* (Figure 5, right panel, 168 *thrC*::pCSK2 (CSK9)). In all cases, a sharp boundary (MIC) was formed, where no cells could grow inside the boundary, but where colonies could be formed in the sub‐MIC concentrations outside the boundary.

Mitomycin C and ciprofloxacin exposure resulted in very similar induction patterns in both *S. aureus* and *B. subtilis* hosts. The colony phenotypes showed a wide toggle‐inducing zone where a varying fraction of cells had switched to a “lytic” (blue) phenotype (top four panels in Figure 5). Hydrogen peroxide showed an extremely thin toggle‐inducing zone for the *S. aureus* host but no toggle‐inducing zone at all for the *B. subtilis* host (bottom panels in Figure 5). The difference in induction pattern most likely reflects that the MTC for hydrogen peroxide is higher than the MIC value in *B. subtilis*, but that MTC and MIC are almost equal in *S. aureus*. For mitomycin C and ciprofloxacin, the MTC value is significantly lower than the MIC value in both hosts.

**FIGURE 6:**
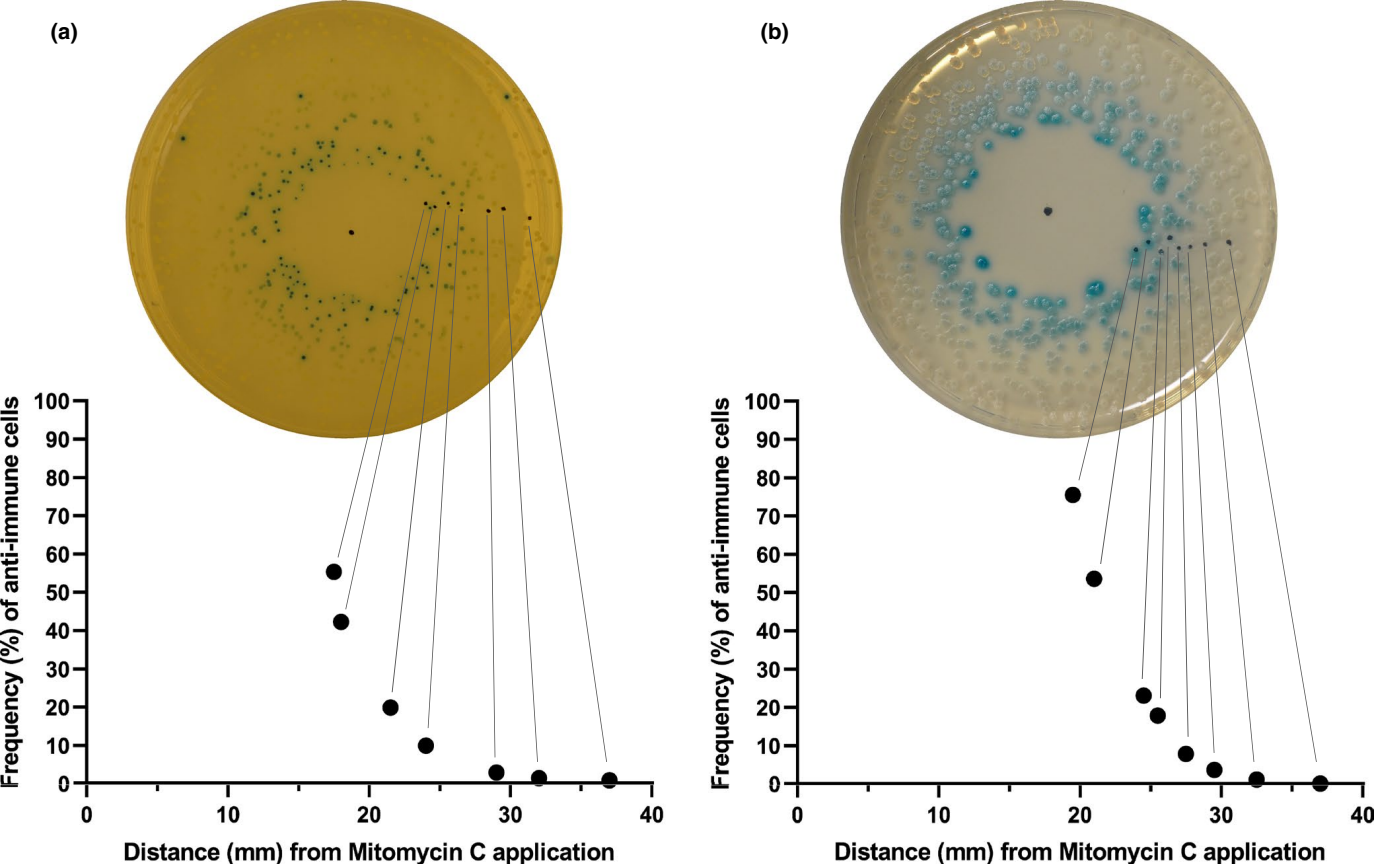
Toggle switching of “lysogenic” cells in response to mitomycin C. Bacteria from a “lysogenic” (white) transformant of (a) 8325‐4 *geh*::pCSK9 (CSK48) and (b) *B. subtilis* 168 *thrC*::pCSK2 (CSK9) were plated, and a small drop of mitomycin C was immediately placed in the center. After growth overnight, individual colonies at different distances to the mitomycin C application spots were picked and suspended in medium without mitomycin C, from which dilutions were plated on LB/TSA X‐gal plates. The frequency of colonies with the “lytic” (blue) phenotype was determined and plotted against the distance to the mitomycin C application. Lines have been inserted to illustrate the relationship between colonies on the plates and the determined frequencies of toggle switching

Across the toggle‐inducing zone of mitomycin C and ciprofloxacin the intensity of blue color decreases (top four panels in Figure 5), likely due to smaller fractions of toggle‐switched cells in the colonies. To quantify the fractions of stable toggle‐switched cells, we picked colonies grown at known distances from an application spot containing mitomycin C and determined the fraction of cells in each colony that could form colonies with a stable “lytic” phenotype. After a suspension of each colony in a fresh medium, plating, incubation, and quantifying the fraction of “lytic” colonies, an exponential dependency was observed between the frequency of “lytic” cells and the distance to the application spot for both *S. aureus* and *B. subtilis* (Figure 6). This could suggest a quasi‐linear dependence between the toggle switch frequency and the mitomycin C concentration since we expect a similar decrease in concentration by diffusion of a compound in agar (Koch, 1999), although the inherent instability of the “lytic” phenotype detected above should be taken into consideration. This dependence of toggle frequency on the concentration of the DNA damaging agent suggests the existence of high stochasticity in either the level of DNA damage, the concentration of RecA* nucleofilaments, the rate of autocleavage of CI, or the competition between the elements in the ɸ13 switch. Experiments to unravel the kinetic relationship between induction of the SOS response and the toggle switching of the genetic switch would be interesting, but it would require simultaneous monitoring of the SOS response.

### 3.9 Timing of commitment to the “lytic” phenotype in *S. aureus*

**FIGURE 7:**
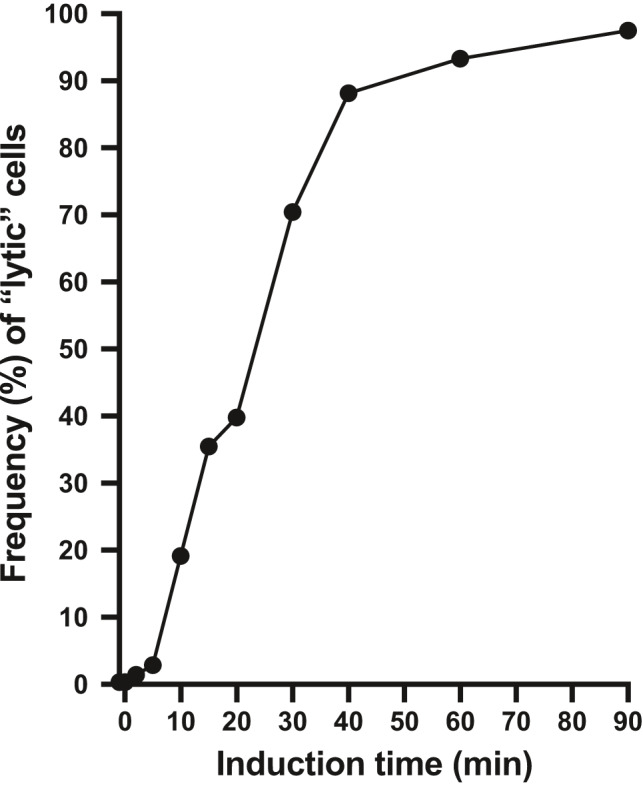
Induction of “lysogenic” to “lytic” toggle switching from pCSK9 plasmids in *S. aureus* by temporal exposure to mitomycin C. “Lysogenic” 8325‐4 *geh*::pCSK9 (CSK48) cells were grown in liquid TSB medium (in duplicates) and induced by addition of 1 µg ml^−1^ mitomycin C at time = 0 min. At the indicated times, aliquots were harvested and dilutions were plated on TSA plates containing X‐gal. The average fraction of colonies (in percent) with the “lytic” phenotype was plotted against the duration (in min) of the exposure to mitomycin C

To examine the timing of the response of the switch to DNA damage, we exposed “lysogenic” 8325‐4 *geh*::pCSK9 (CSK48) cells to mitomycin C at a final concentration of 1 µg ml^−1^. Induction of toggle switch to the “lytic” phenotype was monitored by plating dilutions of the mitomycin C treated culture on TSA plates containing X‐gal. A “lytic” phenotype indicated that the induced cell that formed the colony was committed to the “lytic” phenotype and had passed on the phenotype to the descendants in the colony. Figure 7 shows the temporal increase in the fraction of cells committed to forming colonies with a stable “lytic” phenotype (in percent). The highest frequency of commitment to the “lytic” phenotype was obtained between 5 and 40 min of exposure to mitomycin C, and as many as 30% of all cells became dedicated in 20–30 min. However, because of the stochastic nature of the induction process, a fraction of the bacteria showed commitment in <5 min of exposure to mitomycin C (3% of the cells), while other cells required exposure for more than 40 min before commitment (10% of the cells). As discussed above, the CI threshold allowing toggle switching is determined by many factors, such as the extent of DNA damage, the CI degradation rate, the CI synthesis rate, and ɸ13 switch kinetics, each adding to the overall stochasticity of the process.

## 4 CONCLUSIONS

In this study, we have reported the first analysis of a genetic switch from a Sa3int bacteriophage with implications for the phage‐derived host specificity of *S. aureus* bacteria towards humans. As identical switches are present in other Sa3int phages and unrelated phages carrying genes for PVL, the results are also relevant for a larger group of pathogenic phages. Inhibition of the process leading to phage integration into *S. aureus* hosts could influence their ability to colonize humans. Therefore, it is crucial to understand the process by which the phage switch decides between (i) lysogenization of the host to integrate as a prophage or (ii) multiplying and killing of the host. It is equally important to understand the toggle process that induces a Sa3int prophage to kill the bacterium and release a litter of newly produced phages. We have shown that a minimal genetic switch of ɸ13, contained on a 1.3 kb DNA fragment and expressing functional *cI* and/or *mor* genes from divergent promoters, is fully competent in both decision switching and toggle switching. Purified his‐tagged CI protein was found to be folded, stable, and to bind with high affinity to operators with two half‐sites following the AGTTCAYR consensus, but with low affinity to mutant operators (AGTT **G** AYR). The two alternative phenotypes of the epigenetic switch are not equally stable. While the “lysogenic” phenotype is very stable (although chosen in <5% of the decision events) and leads to the lysogenic life cycle of the phage, the frequent “lytic” phenotype that resembles the first phase in the lytic life cycle is unstable and toggles towards the “lysogenic” phenotype. If this high toggle frequency indicates that prophage induction may be reversible in its early phase, then our preliminary findings that the nutritional status of the cell can modulate the toggle frequency could prove relevant.

Induction of “lysogenic” to “lytic” toggling by DNA damaging agents, resembling the induction of the ɸ13 prophage, was visualized in a plate diffusion assay that enabled us to locate MIC and MTC values (Figure 5). In this work, we defined MTC values as a tool to distinguish between the antibiotic killing of cells by inhibition of cell function from killing by induction of phage lysis. Relative MIC/MTC ratios could be important in the clinical use of DNA damaging antibiotics to prevent phage lysis mediated toxin production (Zhang et al., 2000) or production of host transforming phages like ɸ13. Antibiotics with a MIC/MTC ratio below 1.0 would be safe in this regard; however, as shown for hydrogen peroxide (Figure 5), the MIC/MTC ratio can be strain or species‐specific. Timing of the induction process revealed a highly stochastic process with a maximal toggling rate, leading to dedicated cells with the stable “lytic” phenotype, between 20 and 30 min of exposure to mitomycin C. While the induction of DNA damage in each cell ultimately defines a particular CI degradation rate, this could easily be changing over time due to the SOS induction of DNA repair enzymes. The stochasticity of the toggle frequency at different time points could originate at many levels, including the initial CI and MOR concentrations, their production levels, and the DNA damage‐induced CI degradation rate. While toggle switching has been reported for many bistable switches (Dubnau & Losick, 2006), the ability of an isolated switch to conduct both decision switching and toggle switching has not previously been demonstrated for any phage.

## 5 ETHICS STATEMENT

None required.

## CONFLICT OF INTEREST

None declared.

## AUTHOR CONTRIBUTIONS

**Camilla Schoopp Kristensen:** Conceptualization (equal); Data curation (lead); Investigation (lead); Methodology (equal); Validation (equal); Visualization (equal); Writing‐original draft (equal); Writing‐review & editing (equal). **Anders Kokkenborg Varming:** Data curation (supporting); Formal analysis (supporting); Investigation (supporting); Writing‐review & editing (equal). **Helena Augusta Katharina Leinweber:** Investigation (supporting); Supervision (supporting); Writing‐review & editing (equal). **Karin Hammer:** Conceptualization (supporting); Methodology (supporting); Validation (supporting); Writing‐original draft (supporting); Writing‐review & editing (equal). **Leila Lo Leggio:** Conceptualization (supporting); Data curation (supporting); Formal analysis (supporting); Funding acquisition (equal); Methodology (supporting); Supervision (supporting); Validation (supporting); Writing‐original draft (supporting); Writing‐review & editing (equal). **Hanne Ingmer:** Conceptualization (supporting); Funding acquisition (equal); Supervision (equal); Writing‐original draft (supporting); Writing‐review & editing (equal). **Mogens Kilstrup:** Conceptualization (lead); Data curation (equal); Investigation (equal); Methodology (equal); Project administration (lead); Supervision (lead); Visualization (equal); Writing‐original draft (equal); Writing‐review & editing (equal).

## Data Availability

The data generated and analyzed during the current study are available in the Zenodo repository at https://doi.org/10.5281/zenodo.5515850

## 1

**TABLE A1:** A complete list of bacterial strains used in this study. Strain, species, a short description, and reference of all strains used in this study. See main text for a reference list

Strain	Species	Short description or relevant genotype	Reference or source
168	*B. subtilis*		Burkholder and Giles (1947)
8325‐4	*S. aureus*	NCTC8325 phage cured	Novick (1967)
8325‐4 ɸ13‐kana	*S. aureus*	8325‐4 lysogenized with ɸ13‐kana	Tang et al. (2017)
IM08B	*E. coli*	K12 DH10B *Δdcm hsdMS*	Monk et al. (2015)
CSK8	*B. subtilis*	168 *thrC*::pCSK2 expressing the “lytic” phenotype	This study
CSK9	*B. subtilis*	168 *thrC*::pCSK2 expressing the “lysogenic” phenotype	This study
CSK10	*B. subtilis*	168/pCSK3 expressing the “lytic” phenotype	This study
CSK11	*B. subtilis*	168/pCSK3 expressing the “lysogenic” phenotype	This study
CSK19	*E. coli*	IM08B/pCSK3	This study
CSK26	*E. coli*	IM08B/pCSK9	This study
CSK32	*S. aureus*	8325‐4/pCSK3 expressing the “lytic” phenotype	This study
CSK33	*S. aureus*	8325‐4/pCSK3 expressing the “lysogenic” phenotype	This study
CSK44	*E. coli*	IM08B/pYL112Δ19	This study
CSK45	*S. aureus*	8325‐4/pYL112Δ19	This study
CSK46	*S. aureus*	8325‐4 ɸ13‐kana/pYL112Δ19	This study
CSK47	*S. aureus*	CSK45 *geh*::pCSK9 expressing the “lytic” phenotype	This study
CSK48	*S. aureus*	CSK45 *geh*::pCSK9 expressing the “lysogenic” phenotype	This study
CSK50	*S. aureus*	8325‐4 ɸ13‐kana/pCSK3 expressing the “lysogenic” phenotype	This study
CSK51	*S. aureus*	8325‐4 ɸ13‐kana/pCSK3 expressing the “lytic” phenotype	This study
CSK52	*S. aureus*	8325‐4 ɸ13‐kana *geh*::pCSK9 expressing the “lysogenic” phenotype	This study
CSK54	*E. coli*	IM08B/pCL25	This study
CSK56	*S. aureus*	CSK46 *geh*::pCL25	This study
CSK57	*E. coli*	IM08B/pNZlac	This study
CSK59	*S. aureus*	8325‐4/pNZlac	This study
CSK60	*S. aureus*	8325‐4 ɸ13‐kana/pNZlac	This study
CSK65	*E. coli*	IM08B/pCSK12	This study
CSK67	*S. aureus*	CSK46 *geh*::pCSK12 expressing the “lysogenic” phenotype	This study
CSK68	*S. aureus*	CSK45 *geh*::pCL25	This study
CSK69	*S. aureus*	CSK45 *geh*::pCSK12 expressing the “lysogenic” phenotype	This study
CSK70	*B. subtilis*	168 *thrC*::pCSK11 expressing the “lysogenic” phenotype	This study
CSK77	*E. coli*	IM08B/pCSK13 expressing the “lysogenic” phenotype	This study
CSK78	*S. aureus*	8325‐4/pCSK13 expressing the “lysogenic” phenotype	This study
CSK79	*S. aureus*	8325‐4 ɸ13‐kana/pCSK13 expressing the “lysogenic” phenotype	This study
CSK80	*B. subtilis*	168/pCSK13 expressing the “lysogenic” phenotype	This study

**TABLE A2:** Primers used in this study

Primer name	Sequence (5’ to 3’)
MK740	Cy5‐GTAATACGACTCACTATAGG
MK741	Cy5‐CCTTTTGTGTGAAAGTTACC
MK860	AAAAGGATCCGTGATTTGTTCCATTGTGC
MK861	AAATCTAGATTAAAGCGCTAAATATACGTTATTAATCAC
MK862	AAAAGCATGCGTGATTTGTTCCATTGTGC
MK863	AAAAGAATTCTTAAAGCGCTAAATATACGTTATTAATCAC
MK867	GTAATACGACTCACTATAGGATAATAAAGCTTGTTGAACAAAAATTC
MK868	CCTTTTGTGTGAAAGTTACCACTTTACACCTTGTTTTGAATC
MK869	GTAATACGACTCACTATAGGGACTTGATTCAAAACAAGGTGTAAAG
MK870	CCTTTTGTGTGAAAGTTACCTGAGTAGTCGTAACACATAAAAGC
MK871	GTAATACGACTCACTATAGGATATACCGGTAGAGAAAATACAC
MK872	CCTTTTGTGTGAAAGTTACCGGATCCGTGATTTGTTCCATTGTG
MK902	AAAAAGATCTGCAGACATGGCCTGCCC
MK1038	GTTGAACAAAAATTCAACAAAAAAGTTGATAAATCATCAATTTTTGTATTG
MK1039	CAATACAAAAATTGATGATTTATCAACTTTTTTGTTGAATTTTTGTTCAAC
MK1040	CAAAACAAGGTGTAAAGTATAGTTAAGTTGATGATACGTCAACTTGAGAGGAG
MK1041	CTCCTCTCAAGTTGACGTATCATCAACTTAACTATACTTTACACCTTGTTTTG
MK1042	CACTTATATTTTTTTAAAGAAAAAGTTGATGTTATATCAACTTAAGGAGGGGCAC
MK1043	GTGCCCCTCCTTAAGTTGATATAACATCAACTTTTTCTTTAAAAAAATATAAGTG

Note

Name and sequence of primers used for plasmid construction and gel shift assays. All were primers designed specifically for this study.

**TABLE A3:** Lysogenization frequency after ɸ13‐kana‐infection of *S. aureus* 8325‐4 and comparison to transformation of 8325‐4 by pCKS3

lytic events (PFU ml^−1^)	lysogenic events (CFU(Kan^R^)ml^−1^)	Lysogenization frequency (%)	MOI	Average lysogenization frequency	(p‐value for mean equal to pCSK3)
2.8E+07	2.9E+06	9.4	1	4.6% ± 2.8%	0.87
1.1E+08	2.9E+06	2.6	1		
7.2E+06	3.2E+05	4.3	1		
1.5E+07	7.2E+05	4.6	1		
2.5E+07	6.2E+05	2.4	1		
1.9E+06	1.9E+05	9.1	0.1	3.5% ± 3.3%	0.47
1.2E+07	3.8E+05	3.1	0.1		
8.5E+05	2.1E+04	2.4	0.1		
5.5E+07	4.9E+04	0.1	0.1		
1.1E+07	3.2E+05	2.8	0.1		

Note

*S. aureus* strain 8325‐4 was infected with ɸ13‐kana at MOI at 1 and 0.1, and the frequency of lytic events (phage titer in PFU/ml, column 1) as well as the frequency of lysogenic events (CFU/ml after plating infected cells on TSB plates containing kanamycin, column 2). Column 3 shows the lysogenization frequency for all bacteria infected by ɸ13‐kana (lysogenic events /(lytic events + lysogenic events)) for five biological replicates. Column 4 shows the MOI. Five biological replicates were included for both MOI 1 and MOI 0.1.5. Average lysogenization frequencies are shown in column 5. The p‐values from two‐sample *t*‐tests of ‐average frequency from the infections at MOI of 1 and 0.1 against the frequency observed at transformation of 8325‐4 by pCSK3 (4.9% ± 2.9%) are shown in column 6.

**TABLE A4:** Frequencies of colonies with the “lysogenic” phenotype after the introduction of *mor*‐deficient ɸ13 switch plasmids into *S. aureus* or *B. subtilis* by transformation

*S. aureus* 8325‐4		*S. aureus* 8325‐4 ɸ13‐kana		*B. subtilis* 168	
pCSK12^a^	pCSK13^a^	pCSK12^a^	pCSK13	pCSK11	pCSK13
100.0 ± 0 (99 colonies in total; *n* = 3)	100.0 ± 0 (440 colonies in total; *n* = 6)	100.0 ± 0 (477 colonies in total; *n* = 4)	100.0 ± 0 (*n* = 6)	100.0 ± 0 (*n* = 3)	100.0 ± 0 (*n* = 4)

Note

Switch plasmids were replicative (pCSK13) or required integration into the host (pCSK12 in *S. aureus*, pCSK11 in *B. subtilis*). pCSK11‐13 are *mor*‐deficient versions of pCSK2, pCSK9, and pCSK3, respectively. Recipient bacteria were naïve (*S. aureus* 8325‐4 or *B. subtilis* 168) or ɸ13 lysogens (*S. aureus* 8325‐4 ɸ13‐kana). The number of biological replicates with more than 100 resulting colonies are stated in brackets. Frequencies of “lysogenic” transformant colonies on plates containing X‐gal were calculated as the frequency between “lytic” and “lysogenic” in each transformation event and the average is shown here. Standard deviations are based on differences between each event.

^a^ Indicates that <100 transformants obtained in some or all experiments. The total number of colonies is stated in brackets.

## References

[mbo31245-bib-0001] Altschul, S. F. , Gish, W. , Miller, W. , Myers, E. W. , & Lipman, D. J. (1990). Basic local alignment search tool. Journal of Molecular Biology, 215(3), 403–410. 10.1016/S0022-2836(05)80360-2 2231712

[mbo31245-bib-0002] Atsumi, S. , & Little, J. W. (2006). Role of the lytic repressor in prophage induction of phage λ as analyzed by a module‐replacement approach. Proceedings of the National Academy of Sciences of the United States of America, 103(12), 4558–4563. 10.1073/pnas.0511117103 16537413 PMC1450210

[mbo31245-bib-0003] Burkholder, P. R. , & Giles, N. H. (1947). Induced biochemical mutations in *Bacillus subtilis* . American Journal of Botany, 34(6), 345–348. 10.2307/2437147 20252518

[mbo31245-bib-0004] Casjens, S. R. , & Hendrix, R. W. (2015). Bacteriophage lambda: Early pioneer and still relevant. Virology, 479‐480, 310–330. 10.1016/j.virol.2015.02.010 PMC4424060 25742714

[mbo31245-bib-0005] Coleman, D. , Knights, J. , Russell, R. , Shanley, D. , Birkbeck, T. H. , Dougan, G. , & Charles, I. (1991). Insertional inactivation of the *Staphylococcus aureus* β‐toxin by bacteriophage φ13 occurs by site‐and orientation‐specific integration of the φ13 genome. Molecular Microbiology, 5(4), 933–939. 10.1111/j.1365-2958.1991.tb00768.x 1830359

[mbo31245-bib-0006] Dubnau, D. , & Losick, R. (2006). Bistability in bacteria. Molecular Microbiology, 61(3), 564–572. 10.1111/j.1365-2958.2006.05249.x 16879639

[mbo31245-bib-0007] Gardner, T. S. , Cantor, C. R. , & Collins, J. J. (2000). Construction of a genetic toggle switch in *Escherichia coli* . Nature, 403(6767), 339–342. 10.1038/35002131 10659857

[mbo31245-bib-0008] Gasteiger, E. , Hoogland, C. , Gattiker, A. , Duvaud, S. , Wilkins, M. R. , Appel, R. D. , & Bairoch, A. (2005). Protein identification and analysis tools on the ExPASy server. In J. M. Walker (Eds.), The proteomics protocols handbook (pp. 571–608). Humana Press.

[mbo31245-bib-0009] Goerke, C. , Koller, J. , & Wolz, C. (2006). Ciprofloxacin and trimethoprim cause phage induction and virulence modulation in *Staphylococcus aureus* . Antimicrobial Agents and Chemotherapy, 50(1), 171–177. 10.1128/AAC.50.1.171-177.2006 16377683 PMC1346766

[mbo31245-bib-0010] Goerke, C. , Pantucek, R. , Holtfreter, S. , Schulte, B. , Zink, M. , Grumann, D. , Bröker, B. M. , Doskar, J. , & Wolz, C. (2009) Diversity of prophages in dominant *Staphylococcus aureus* clonal lineages. Journal of Bacteriology, 191(11), 3462–3468. 10.1128/JB.01804-08 19329640 PMC2681900

[mbo31245-bib-0011] Goerke, C. , Wirtz, C. , Fluckiger, U. , & Wolz, C. (2006). Extensive phage dynamics in *Staphylococcus aureus* contributes to adaptation to the human host during infection. Molecular Microbiology, 61(6), 1673–1685. 10.1111/j.1365-2958.2006.05354.x 16968231

[mbo31245-bib-0012] Grodzicker, T. , Arditti, R. R. , & Eisen, H. (1972). Establishment of repression by lambdoid phage in catabolite activator protein and adenylate cyclase mutants of *Escherichia coli* . Proceedings of the National Academy of Sciences of the United States of America, 69(2), 366–370. 10.1073/pnas.69.2.366 4333980 PMC426459

[mbo31245-bib-0013] Guérout‐Fleury, A. M. , Frandsen, N. , & Stragier, P. (1996). Plasmids for ectopic integration in *Bacillus subtilis* . Gene, 180(1–2), 57–61. 10.1016/S0378-1119(96)00404-0 8973347

[mbo31245-bib-0014] Herman, C. , Ogura, T. , Tomoyasu, T. , Hiraga, S. , Akiyama, Y. , Ito, K. , Thomas, R. , D'Ari, R. , & Bouloc, P. (1993). Cell growth and λ phage development controlled by the same essential *Escherichia coli* gene, *ftsH/hflB* . Proceedings of the National Academy of Sciences of the United States of America, 90(22), 10861–10865. 10.1073/pnas.90.22.10861 8248182 PMC47878

[mbo31245-bib-0015] Huseby, M. , Shi, K. , Brown, C. K. , Digre, J. , Mengistu, F. , Seo, K. S. , Bohach, G. A. , Schlievert, P. M. , Ohlendorf, D. H. , & Earhart, C. A. (2007). Structure and biological activities of beta toxin from *Staphylococcus aureus* . Journal of Bacteriology, 189(23), 8719–8726. 10.1128/JB.00741-07 17873030 PMC2168928

[mbo31245-bib-0016] Ingmer, H. , Gerlach, D. , & Wolz, C. (2019). Temperate phages of *Staphylococcus aureus* . Microbiology Spectrum, 7(5), 10.1128/microbiolspec.gpp3-0058-2018 PMC10921950 31562736

[mbo31245-bib-0017] Johansen, A. H. , Brøndsted, L. , & Hammer, K. (2003). Identification of operator sites of the CI repressor of phage TP901‐1: Evolutionary link to other phages. Virology, 311(1), 144–156. 10.1016/S0042-6822(03)00169-7 12832212

[mbo31245-bib-0018] Katayama, Y. , Baba, T. , Sekine, M. , Fukuda, M. , & Hiramatsu, K. (2013). Beta‐hemolysin promotes skin colonization by *Staphylococcus aureus* . Journal of Bacteriology, 195(6), 1194–1203. 10.1128/JB.01786-12 23292775 PMC3592002

[mbo31245-bib-0019] Kihara, A. , Akiyama, Y. , & Ito, K. (2001). Revisiting the lysogenization control of bacteriophage λ. Identification and characterization of a new host component, HflD. Journal of Biological Chemistry, 276(17), 13695–13700. 10.1074/jbc.M011699200 11278968

[mbo31245-bib-0020] Koch, A. L. (1999). Diffusion through agar blocks of finite dimensions: A theoretical analysis of three systems of practical significance in microbiology. Microbiology, 145(3), 643–654. 10.1099/13500872-145-3-643 10217498

[mbo31245-bib-0021] Konkol, M. A. , Blair, K. M. , & Kearns, D. B. (2013). ‘Plasmid‐encoded ComI inhibits competence in the ancestral 3610 strain of *Bacillus subtilis* . Journal of Bacteriology, 195(18), 4085–4093. 10.1128/JB.00696-13 23836866 PMC3754741

[mbo31245-bib-0022] Kovács, A. T. , van Hartskamp, M. , Kuipers, O. P. , & van Kranenburg, R. (2010). Genetic tool development for a new host for biotechnology, the thermotolerant bacterium *Bacillus coagulans* . Applied and Environmental Microbiology, 76(12), 4085–4088. 10.1128/AEM.03060-09 20400555 PMC2893497

[mbo31245-bib-0023] Lee, C. Y. , Buranen, S. L. , & Zhi‐Hai, Y. (1991). Construction of single‐copy integration vectors for *Staphylococcus aureus* . Gene, 103(1), 101–105. 10.1016/0378-1119(91)90399-V 1652539

[mbo31245-bib-0024] Little, J. W. (1984). Autodigestion of *lexA* and phage λ repressors. Proceedings of the National Academy of Sciences of the United States of America, 81(3I), 1375–1379. 10.1073/pnas.81.5.1375 6231641 PMC344836

[mbo31245-bib-0025] Liu, G. Y. (2009). Molecular pathogenesis of *Staphylococcus aureus* infection. Pediatric Research, 65, 71R–77R. 10.1203/PDR.0b013e31819dc44d PMC2919328 19190527

[mbo31245-bib-0026] Lowy, F. D. (1998). *Staphylococcus aureus* infections. New England Journal of Medicine, 339(8), 520–532. 10.1056/NEJM199808203390806 9709046

[mbo31245-bib-0027] Luong, T. T. , & Lee, C. Y. (2007). Improved single‐copy integration vectors for *Staphylococcus aureus* . Journal of Microbiological Methods, 70(1), 186–190. 10.1016/j.mimet.2007.04.007 17512993 PMC2001203

[mbo31245-bib-0028] Madsen, P. L. , Johansen, A. H. , Hammer, K. , & Brøndsted, L. (1999). ‘The genetic switch regulating activity of early promoters of the temperate lactococcal bacteriophage TP901‐1. Journal of Bacteriology, 181(24), 7430–7438. 10.1128/jb.181.24.7430-7438.1999 10601198 PMC94198

[mbo31245-bib-0029] Micsonai, A. , Wien, F. , Bulyáki, É. , Kun, J. , Moussong, É. , Lee, Y.‐H. , Goto, Y. , Réfrégiers, M. , & Kardos, J. (2018). BeStSel: A web server for accurate protein secondary structure prediction and fold recognition from the circular dichroism spectra. Nucleic Acids Research, 46(W1), W315–W322. 10.1093/nar/gky497 29893907 PMC6031044

[mbo31245-bib-0030] Micsonai, A. , Wien, F. , Kernya, L. , Lee, Y.‐H. , Goto, Y. , Réfrégiers, M. , & Kardos, J. (2015). Accurate secondary structure prediction and fold recognition for circular dichroism spectroscopy. Proceedings of the National Academy of Sciences of the United States of America, 112(24), E3095–E3103. 10.1073/pnas.1500851112 26038575 PMC4475991

[mbo31245-bib-0031] Monk, I. R. , Tree, J. J. , Howden, B. P. , Stinear, T. P. , & Foster, T. J. (2015). Complete bypass of restriction systems for major *Staphylococcus aureus* lineages. MBio, 6(3), 1–12. 10.1128/mBio.00308-15 PMC4447248 26015493

[mbo31245-bib-0032] Novick, R. P. (1963). Analysis by transduction of mutations affecting penicillinase formation. Journal of General Microbiology, 33(1), 121–136. 10.1099/00221287-33-1-121 14072829

[mbo31245-bib-0033] Novick, R. (1967). Properties of a cryptic high‐frequency transducing phage in *Staphylococcus aureus* . Virology, 33(1), 155–166. 10.1016/0042-6822(67)90105-5 4227577

[mbo31245-bib-0034] Oliveira, H. , Sampaio, M. , Melo, L. D. R. , Dias, O. , Pope, W. H. , Hatfull, G. F. , & Azeredo, J. (2019). Staphylococci phages display vast genomic diversity and evolutionary relationships. BMC Genomics, 20, 357. 10.1186/s12864-019-5647-8 31072320 PMC6507118

[mbo31245-bib-0035] Oppenheim, A. B. , Kobiler, O. , Stavans, J. , Court, D. L. , & Adhya, S. (2005). Switches in bacteriophage lambda development. Annual Review of Genetics, 39(1), 409–429. 10.1146/annurev.genet.39.073003.113656 16285866

[mbo31245-bib-0036] Pedersen, M. , & Hammer, K. (2008). The role of MOR and the CI operator sites on the genetic switch of the temperate bacteriophage TP901‐1. Journal of Molecular Biology, 384(3), 577–589. 10.1016/j.jmb.2008.09.071 18930065

[mbo31245-bib-0037] Pedersen, M. , Neergaard, J. T. , Cassias, J. , Rasmussen, K. K. , Lo Leggio, L. , Sneppen, K. , Hammer, K. , & Kilstrup, M. (2020). Repression of the lysogenic PR promoter in bacteriophage TP901‐1 through binding of a CI‐MOR complex to a composite OM‐OR operator. Scientific Reports, 10(1), 8659. 10.1038/s41598-020-65493-0 32457340 PMC7250872

[mbo31245-bib-0038] Riva, S. , Polsinelli, M. , & Falaschi, A. (1968). A new phage of *Bacillus subtilis* with infectious DNA having separable strands. Journal of Molecular Biology, 35(2), 347–356. 10.1016/S0022-2836(68)80029-4 5000316

[mbo31245-bib-0039] Roucourt, B. , & Lavigne, R. (2009). The role of interactions between phage and bacterial proteins within the infected cell: A diverse and puzzling interactome. Environmental Microbiology, 11(11), 2789–2805. 10.1111/j.1462-2920.2009.02029.x 19691505

[mbo31245-bib-0040] Salgado‐Pabón, W. , Herrera, A. , Vu, B. G. , Stach, C. S. , Merriman, J. A. , Spaulding, A. R. , & Schlievert, P. M. (2014). *Staphylococcus aureus* β‐toxin production is common in strains with the β‐toxin gene inactivated by bacteriophage. Journal of Infectious Diseases, 210(5), 784–792. 10.1093/infdis/jiu146 PMC4202305 24620023

[mbo31245-bib-0041] Schindelin, J. , Arganda‐Carreras, I. , Frise, E. , Kaynig, V. , Longair, M. , Pietzsch, T. , Preibisch, S. , Rueden, C. , Saalfeld, S. , Schmid, B. , Tinevez, J.‐Y. , White, D. J. , Hartenstein, V. , Eliceiri, K. , Tomancak, P. , & Cardona, A. (2012). Fiji: An open‐source platform for biological‐image analysis. Nature Methods, 9(7), 676–682. 10.1038/nmeth.2019 22743772 PMC3855844

[mbo31245-bib-0042] Sreerama, N. , & Woody, R. W. (2000). Estimation of protein secondary structure from circular dichroism spectra: Comparison of CONTIN, SELCON, and CDSSTR methods with an expanded reference set. Analytical Biochemistry, 287(2), 252–260. 10.1006/abio.2000.4880 11112271

[mbo31245-bib-0043] Tang, Y. , Nielsen, L. N. , Hvitved, A. , Haaber, J. K. , Wirtz, C. , Andersen, P. S. , Larsen, J. , Wolz, C. , & Ingmer, H. (2017). Commercial biocides induce transfer of prophage Φ13 from human strains of *Staphylococcus aureus* to livestock CC398. Frontiers in Microbiology, 8, 2418. 10.3389/fmicb.2017.02418 29270158 PMC5726172

[mbo31245-bib-0044] Tong, S. Y. C. , Davis, J. S. , Eichenberger, E. , Holland, T. L. , & Fowler, V. G. (2015). *Staphylococcus aureus* infections: Epidemiology, pathophysiology, clinical manifestations, and management. Clinical Microbiology Reviews, 28(3), 603–661. 10.1128/CMR.00134-14 26016486 PMC4451395

[mbo31245-bib-0045] Whitmore, L. , & Wallace, B. A. (2008). Protein secondary structure analyses from circular dichroism spectroscopy: Methods and reference databases. Biopolymers, 89(5), 392–400. 10.1002/bip.20853 17896349

[mbo31245-bib-0046] Xia, G. , & Wolz, C. (2014). Phages of *Staphylococcus aureus* and their impact on host evolution. Infection, Genetics and Evolution, 21, 593–601. 10.1016/j.meegid.2013.04.022 23660485

[mbo31245-bib-0047] Zhang, X. , McDaniel, A. D. , Wolf, L. E. , Keusch, G. T. , Waldor, M. K. , & Acheson, D. W. K. (2000). Quinolone antibiotics induce Shiga toxin‐encoding bacteriophages, toxin production, and death in mice. The Journal of Infectious Diseases, 181(2), 664–670. 10.1086/315239 10669353

